# Dendrimer-based selective autophagy-induction rescues ΔF508-CFTR and inhibits *Pseudomonas aeruginosa* infection in cystic fibrosis

**DOI:** 10.1371/journal.pone.0184793

**Published:** 2017-09-13

**Authors:** Scott Mackenzie Brockman, Manish Bodas, David Silverberg, Ajit Sharma, Neeraj Vij

**Affiliations:** 1 College of Medicine, Central Michigan University, Mount Pleasant, Michigan, United States of America; 2 Department of Chemistry and Biochemistry, Central Michigan University, Mount Pleasant, Michigan, United States of America; 3 Department of Pediatric Respiratory Sciences, The Johns Hopkins School of Medicine, Baltimore, Maryland, United States of America; Universidad de Castilla-La Mancha, SPAIN

## Abstract

**Background:**

Cystic Fibrosis (CF) is a genetic disorder caused by mutation(s) in the CF-transmembrane conductance regulator (*Cftr*) gene. The most common mutation, ΔF508, leads to accumulation of defective-CFTR protein in aggresome-bodies. Additionally, *Pseudomonas aeruginosa* (*Pa*), a common CF pathogen, exacerbates obstructive CF lung pathology. In the present study, we aimed to develop and test a novel strategy to improve the bioavailability and potentially achieve targeted drug delivery of cysteamine, a potent autophagy-inducing drug with anti-bacterial properties, by developing a dendrimer (PAMAM-DEN)-based cysteamine analogue.

**Results:**

We first evaluated the effect of dendrimer-based cysteamine analogue (PAMAM-DEN^CYS^) on the intrinsic autophagy response in IB3-1 cells and observed a significant reduction in Ub-RFP and LC3-GFP co-localization (aggresome-bodies) by PAMAM-DEN^CYS^ treatment as compared to plain dendrimer (PAMAM-DEN) control. Next, we observed that PAMAM-DEN^CYS^ treatment shows a modest rescue of ΔF508-CFTR as the C-form. Moreover, immunofluorescence microscopy of HEK-293 cells transfected with ΔF508-CFTR-GFP showed that PAMAM-DEN^CYS^ is able to rescue the misfolded-ΔF508-CFTR from aggresome-bodies by inducing its trafficking to the plasma membrane. We further verified these results by flow cytometry and observed significant (p<0.05; PAMAM-DEN vs. PAMAM-DEN^CYS^) rescue of membrane-ΔF508-CFTR with PAMAM-DEN^CYS^ treatment using non-permeabilized IB3-1 cells immunostained for CFTR. Finally, we assessed the autophagy-mediated bacterial clearance potential of PAMAM-DEN^CYS^ by treating IB3-1 cells infected with PA01-GFP, and observed a significant (p<0.01; PAMAM-DEN vs. PAMAM-DEN^CYS^) decrease in intracellular bacterial counts by immunofluorescence microscopy and flow cytometry. Also, PAMAM-DEN^CYS^ treatment significantly inhibits the growth of PA01-GFP bacteria and demonstrates potent mucolytic properties.

**Conclusions:**

We demonstrate here the efficacy of dendrimer-based autophagy-induction in preventing sequestration of ΔF508-CFTR to aggresome-bodies while promoting its trafficking to the plasma membrane. Moreover, PAMAM-DEN^CYS^ decreases *Pa* infection and growth, while showing mucolytic properties, suggesting its potential in rescuing *Pa*-induced ΔF508-CF lung disease that warrants further investigation in CF murine model.

## Introduction

The cystic fibrosis transmembrane conductance regulator (CFTR) is a chloride channel found on epithelial cell membranes [1–10], and its dysfunction is associated with cystic fibrosis (CF) that involves dysregulation of epithelial fluid transport in the lungs, pancreas, and other organs of the body [7, 8, 11, 12]. Mutations in the *Cftr* gene (most common being the ΔF508) is characterized by build-up of thick mucus, frequent respiratory infections (such as *Pseudomonas aeruginosa*, *Pa*) and inflammation leading to severe lung damage [7–9, 13–17]. These pathological manifestations caused by dysfunctional CFTR potentially involve autophagy-impairment as an important cellular mechanism associated with pathogenesis of CF lung disease [8, 18, 19]. Autophagy is a host protective mechanism utilized by the cell to sequester and degrade components of the cytosol within double membrane bound vesicles termed autophagosomes [2, 7, 8, 19–21]. Previous studies have demonstrated that ΔF508-CFTR impairs autophagy by activating transglutaminase 2 (TGM2) causing subsequent crosslinking of beclin 1 (BECN1), a necessary protein for autophagy [7, 8]. Additionally, misfolding of ΔF508-CFTR protein can activate reactive oxygen species (ROS) inducing cellular inflammatory-oxidative stress responses, which can further impair autophagy [8]. We anticipate that ROS activation and resulting autophagy-impairment is induced by accumulation of misfolded ΔF508-CFTR protein in perinuclear aggresome-bodies that not only leads to membrane CFTR-dysfunction but also chronic inflammation, initiating the pathogenesis of chronic obstructive CF lung disease.

Cysteamine, the reduced form of cystamine, is an FDA approved drug that has anti-oxidant, anti-biofilm, and mucolytic properties [7, 12, 15, 22]. This drug has been shown to decrease lung inflammation and improve lung function in CF patients in a recent clinical trial, by restoring autophagy and allowing mature-CFTR protein to be trafficked to the plasma membrane (PM) [7, 8, 23]. Cysteamine is an inhibitor of TGM2, and thus increases the amount of BECN1 that is necessary for restoration of autophagy. Furthermore, cysteamine’s anti-oxidant properties can decrease intracellular ROS levels thus promoting autophagy restoration, and forward trafficking of ΔF508-CFTR from ER or aggresome-bodies to the PM.

It has been reported that 50% of CF patients under the age of 18 are infected by *Pa*, with its prevalence significantly increased to 80% in patients over that age [13]. Furthermore, lung infection with *Pa* correlates clinically with impaired lung function and increased morbidity and mortality [1, 10, 12–17, 20]. The biofilms formed by *Pa* facilitates its adherence to lung epithelial cells and contributes to antibiotic resistance, compounding the difficulty of treating CF patients with chronic *Pa* infection [17]. Until recently, *Pa* was considered to be an extracellular pathogen, although it has been shown that *Pa* has the ability to gain access and reside within the host cells as an intracellular pathogen. Thus, bronchial epithelial cells provide a repository of this pathogen during chronic *Pa* infection [20]. The clearance of these intracellular pathogens is mediated by autophagy [12], and we hypothesize that its dysfunction can impair the ability of CF cells to clear chronic infection. Cysteamine, which also induces autophagy, has been reported to have a direct bactericidal potential against *Pa* through its anti-biofilm and anti-microbial properties [12]. Moreover, a recent study shows that cysteamine has the potential to induce *Pa*-clearance by CF-macrophages that involves up-regulation of pro-autophagy protein, Beclin-1, and re-establishment of the autophagy process [24]. We anticipate that cysteamine’s autophagy-inducing property greatly enhances its therapeutic potential both as a CFTR corrector and an anti-bacterial, thus warranting its further pharmacological development for treating chronic stages of CF lung disease.

Additionally, chronic inflammation and unabated mucus production in CF provides a physical barrier for efficient drug delivery [14, 15, 25]. Thus, nanoparticle-based drug delivery approach has become a compelling choice for efficacious therapeutic intervention in CF [11, 14, 15, 25]. Polyamidoamine (PAMAM) dendrimers have been extensively studied and have shown promise as a sustained and targeted drug delivery system [26, 27]. Hence, in this study, we modified the terminal group of a cationic dendrimer with terminal amine groups to a “cysteamine-like structure with a sulfhydryl group” in order to take advantage of its known mucolytic and anti-bacterial properties for effective delivery from airway lumen to target epithelial cells [12]. Based on the known properties of cysteamine, we postulate that our novel PAMAM-DEN^CYS^ formulation could potentially target chronic inflammatory-oxidative stress (anti-oxidant), impaired-autophagy (autophagy-inducer) and *Pa-* growth (anti-bacterial) in CF lungs [12, 28]. In addition to cysteamine, PAMAM dendrimers (PAMAM-DEN) have also been shown to possess some anti-oxidant potential by themselves, thus allowing control of ROS mediated autophagy-impairment in CF [26, 27, 29, 30].

Overall, we not only verify here the role of impaired autophagy in sequestering ΔF508-CFTR to perinuclear aggresome-bodies but also demonstrate the efficacy of our novel nano-formulation, PAMAM-DEN^CYS^, in promoting ΔF508-CFTR trafficking to the PM. Moreover, PAMAM-DEN^CYS^ mediated autophagy also decreases *Pa* infection in CF cells, suggesting its therapeutic potential in rescuing *Pa*-induced ΔF508-CF lung disease. Thus, our data provides substantial preliminary evidence that warrants further investigation of this cysteamine analogue-formulation in pre-clinical models of CF lung disease.

## Materials and methods

### Cell culture conditions, transfection and treatments

CFBE41o-/IB3-1 cells were cultured in MEM media supplemented with 10% fetal bovine serum (FBS), 1% Penicillin, Streptomycin and Amphotericin (PSA) and 1% Glutamine (200 mM stock concentration, Gibco/ThermoFisher) and maintained at 37°C/5% CO_2_ atmosphere. The PSA solution contains 10,000 Units/ml of penicillin, 10,000 μg/ml of streptomycin and 25 μg/ml amphotericin, as per the company’s item description (PSA, Gibco/ThermoFisher). The T-75 flasks were coated with fibronectin solution composed of LHC basal media, 10% bovine serum albumin (BSA), 1% collagen I and 1% fibronectin to facilitate epithelial cell adhesion. When treating with dendrimers, the final concentrations of Dendrimer-cysteamine (PAMAM-DEN^CYS^) alone were set to the indicated cysteamine concentration and equal volume of dendrimer were used as control. HEK-293 cells cultured in DMEM/F-12 media supplemented with 10% FBS and 1% PSA, were transiently transfected with ΔF508-CFTR-GFP plasmid using Lipofectamine^™^ 2000 reagent (Invitrogen). After 36 hours, cells were treated with either empty dendrimer (PAMAM-DEN, 500 μM) or PAMAM-DEN^CYS^ (500 μM) for 12 hours. Images were captured using ZOE^™^ Fluorescent Cell Imager.

### Bacterial culture conditions, infection and clearance

*Pseudomonas aeruginosa*-GFP (PA01-GFP) bacteria were grown overnight at 37°C in a shaker incubator at 200 rpm in LB media supplemented with 1% carbenicillin (100 μg/ml; Gibco/ThermoFisher). Next day, optical density was measured using spectrophotometry to calculate multiplicity of infection (MOI) as described before [10]. The IB3-1 cells were pretreated with PAMAM-DEN or PAMAM-DEN^CYS^ for 12 hours, followed by PaO1-GFP infection at MOI of 1 or 10 for 3 hours. In order to quantify the number of intracellular bacteria, the cells were thoroughly washed with PBS (1x) to remove any extracellular bacteria. The images were captured using ZOE^™^ Fluorescent Cell Imager and analysis was done by counting PA01-GFP positive cells. In a separate experiment, IB3-1 cells were transiently transfected with WT-CFTR (24 hours) and pretreated for 12 hours with PAMAM-DEN or PAMAM-DEN^CYS^, before adding PaO1-GFP at MOI of 1 for 3 hours, followed by florescence microscopy as described above.

For assessing the direct bactericidal efficacy of the nano-formulation, *PaO1* bacteria were incubated in LB broth with either control (PBS), PAMAM-DEN (500 μM), cysteamine (500 μM) or PAMAM-DEN^CYS^ (500 μM) at 37°C. The standard growth of bacteria during the logarithmic phase was monitored from zero to 18 hours by measuring the OD (625 nm) of the bacterial culture at every 3 hours interval. The percentage change in bacterial growth was plotted against increasing time [12]. Next, the impact of PAMAM-DEN^CYS^ (500 μM) on individual mucus components (mucin) was evaluated by quantifying the changes in viscoelasticity of sterile 5% w/v porcine mucin solution as compared to control (PBS), PAMAM-DEN and cysteamine (500 μM). The changes in viscosity were quantified by distance travelled (mm) as a function of time (sec) through a sterile 1 ml serological pipette [12].

### Synthesis of G4 PAMAM dendrimer with sulfhydryl groups

**Fig 1:**
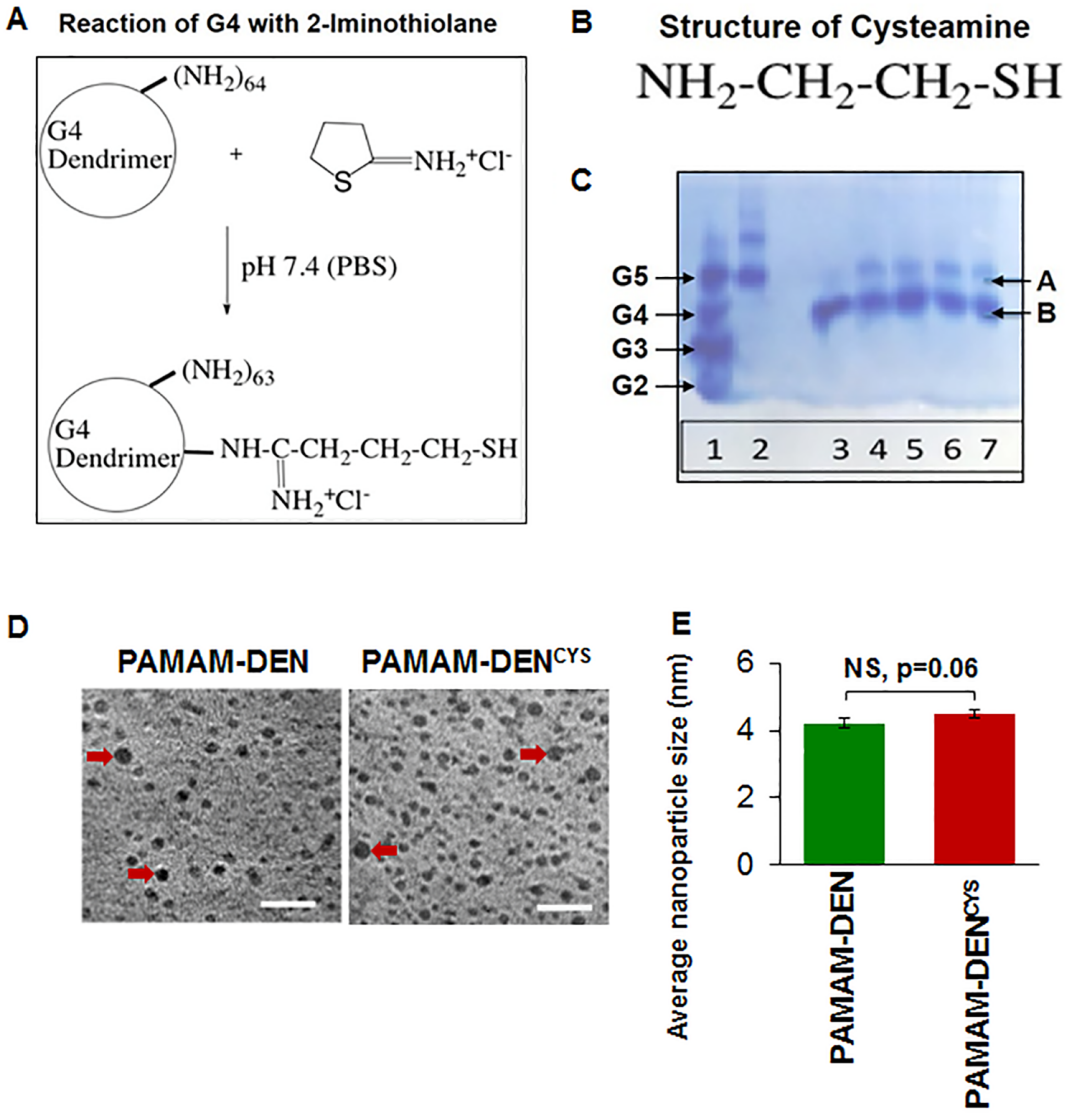
Synthesis and characterization of dendrimer-cysteamine nano-formulation. (A) The schematic shows the reaction for the synthesis of PAMAM generation 4 (G4) dendrimers with terminal end decorated with a cysteamine-analogue. (B) Illustration showing the chemical structure of cysteamine. (C) Native PAGE gel analysis showing the characterization of our nano-formulation, where ‘band B’ represents the dendrimer alone (or dendrimer with one sulfhydryl group) and the slower migrating ‘band A’ depicts dendrimers with a few–SH groups. The different lanes represent the following: Lane 1 –ladder (G2-G5), Lane 2 –G5; Lane 3 –Untreated G4; Lane 4 –Nanoconjugate obtained after synthesis; Lane 5 –the synthesis reaction performed for 24 hrs; Lane 6–7 –the conjugate stored for 2 months showing that there is no cross linking of the conjugates by difulfide formation. The data shows the successful synthesis of a novel PAMAM-DEN^CYS^ nano-formulation for evaluation of its efficacy in CF. (D) Transmission electron microscopy (TEM) images were captured to determine the dispersion and size of the empty (PAMAM-DEN) and cysteamine-conjugated dendrimers PAMAM-DEN^CYS^ (Scale bar: 20 nm). Data shows the clear dispersion of PAMAM-DEN/PAMAM-DEN^CYS^, without any significant aggregation (red arrows showing an example of aggregation). (E) Next, QUARTZ PCI TEM analysis software was used to quantify the average (mean ± SEM) diameter of the dendrimers (DEN/DEN^CYS^), which was determined to be ~4nm (NS = not significant).

PAMAM Generation 4 (G4) diaminobutane (DAB) core with amine surface was synthesized as previously reported [31]. Next, G4-SH was formed by reacting G4 DAB core amine dendrimer (0.7 μmol dendrimer, which is equivalent to 45 μmol NH_2_) with 4.3 μmol 2-Iminothiolane.HCl (Traut’s reagent). This reaction was performed in phosphate-buffered saline (PBS) for 1 hour at room temperature. Finally, the reaction mixture was washed with PBS and filtered using a 3K centrifugal device (Nanosep 3K Omega, Pall Life Sciences). It was stored frozen and thawed before use. The reaction schema is shown in Fig 1A together with the chemical structure of cysteamine (Fig 1B).

### Characterization of G4 PAMAM dendrimer with sulfhydryl groups and transmission electron microscopy

Dendrimers reacted with Traut’s reagent were run on acidic PAGE gels (10% stacking; 10% resolving) as previously described [32]. Each lane contained 5 μg dendrimer or conjugate. Dendrimer and conjugate bands were visualized with Coomassie Blue staining. The concentration of sulfhydryl groups was quantified using Ellman’s reagent [5,5’-dithio-bis-(2-nitrobenzoic acid)]. Briefly, 50 μl of sample (conjugate) was mixed with 950 μl of Ellman’s reagent in a cuvette and the absorbance was measured at 412 nm after two minutes. The concentration of the free -SH groups in the conjugate was calculated based on the absorbance value. Next, the concentration of dendrimers in 50 μl of sample was calculated using the molecular weight (MW) of dendrimer. Finally, the concentration of free -SH was divided by concentration of dendrimers to obtain the approximate number of -SH groups attached per dendrimer. We also used Transmission electron microscopy (TEM) to determine the dendrimer size and shape. Briefly, dendrimers were drop-coated on a carbon-coated copper grid for size measurement, and analysis was performed as recently described [14, 33].

### Immunoblotting

CFBE41o- cells were cultured on six well plates with either control (PBS), PAMAM-DEN, PAMAM-DEN^CYS^ (500μM) or cysteamine alone (500uM). After 48 hours treatment, whole cell protein extracts were collected by adding RIPA buffer, supplemented with 0.5 M EDTA and 1x Halt^™^ Protease inhibitor cocktail (Thermo Fisher) to each well. Isolated total proteins were separated using 7.5% SDS-PAGE gel and immunoblotted onto nitrocellulose membrane. The CFTR 181 antibody [34–36] and β-actin (equal loading control, Sigma, 1:10,000) antibodies were used as primary antibodies, while donkey anti-rabbit IgG HRP and goat anti-mouse IgG HRP was used as a secondary antibodies (1:10000 and 1:6000 respectively, Amersham). Membranes were visualized using the Clarity^™^ Western ECL Blotting substrate (Bio-Rad) and C-DiGit Blot Scanner (LI-COR). Quantifiable changes in protein expression were analyzed using the ImageJ Studio Digits 4.0 software.

### Autophagy reporter assay

To visualize autophagy-impairment (LC3B/Ubiquitin co-localization), IB3-1 cells were transiently-transfected with LC3-GFP and ubiquitin-RFP plasmid constructs using the Lipofectamine^™^ 2000 reagent (Invitrogen) for 24 hours as we recently described [18]. After 24 hours, cells were treated with either PAMAM-DEN or PAMAM-DEN^CYS^ (500μM) for 24 hours. Images were captured using the ZOE^™^ Fluorescent Cell Imager.

### Flow cytometry

IB3-1 cells were treated with either PAMAM-DEN or PAMAM-DEN^CYS^ (500μM) for 12 hours. After treatment, cells were washed (1x) with ice-cold PBS, fixed in 4% paraformaldehyde (PFA) for 15 mins and stained with CFTR (Santa Cruz; 1μg/ml) antibody for 30 mins on ice. Next, cells were washed with ice-cold PBS (1x) and incubated with donkey anti-rabbit IgG-CFL-488 secondary antibody for 20 mins. These non-permeabilized cells were washed with PBS (1x) and re-suspended in 0.1%-paraformaldehyde, followed by acquisition and analysis of data to detect changes in membrane-CFTR levels, using the BD FACSAria flow cytometer and BD FACS Diva software. In a separate experiment, PAMAM-DEN and PAMAM-DEN^CYS^ treated cells infected with *PaO1*-GFP (MOI: 1) bacteria, were washed in PBS (1x) to remove all extracellular bacteria, fixed and permeabilized using BD Fix and PERM reagent, and analyzed by flow cytometry as described above to quantify the changes in total number of intracellular bacteria.

### Statistical analysis

Data is represented as mean ± SEM of at least three independent or parallel experimental replicates. Significance was calculated using a two-tailed unpaired t-test. A p-value of less than 0.05 was considered significant. Densitometry was performed using the Image Studio Digits 4.0 software program as described previously [37]. The densitometry values obtained for the ‘non-treated control’ or ‘PAMAM-DEN’ groups were converted to 100%, and the corresponding values for the ‘cysteamine’ or PAMAM-DEN^CYS^ treatment group were calculated and plotted as percentage change from appropriate control as indicated.

## Results

### Synthesis and characterization of cysteamine-dendrimers

As described in Methods, reaction of G4 DAB core amine dendrimer (0.7 μmol dendrimer, which is equivalent to 45 μmol NH_2_) with 4.3 μmol 2-Iminothiolane.HCl (Traut’s reagent) resulted in an average of one sulhydryl group attached to each dendrimer (Fig 1A and 1B) based on the Ellman’s assay for–SH groups. Ellman’s test was performed as described in the methods. The concentration of the free -SH groups in the in the sample was calculated to be (50 μl x 51pmol/μl) 2550 pmol while the concentration of dendrimers in the same sample (50 μl) was 50 /14243 (molecular weight) = 0.0035 μmol (or 3510 pmol). Using these two values, the number of -SH groups attached per dendrimer was determined to be (2550 pmol SH / 3510 pmol dendrimer = 0.73) approximately one. The acidic PAGE was used to analyze these conjugates (Fig 1C) and unreacted G4 (MW 14243 Da) showed only one band “B” (lane 3). For comparison, ladder (G2-G5) is shown in lane 1 and a G5 is shown in lane 2. Lane 4 shows the conjugate obtained under our synthesis conditions. A major band “B” likely represents the dendrimer alone or dendrimer with one sulfhydryl group since the molecular weight or charge does not change much for such conjugates. The slower migrating minor band “A” probably represents dendrimers with a few–SH groups attached to each nanomolecule. Reaction performed for 24 hours (lane 5) also showed similar results. Lanes 6 and 7 represent conjugates stored frozen for 2 months. These conjugates also showed two bands similar to freshly prepared conjugates. Thus, there is no evidence of cross-linking of the conjugates *via* disulfide formation when stored frozen. The size and dispersion of PAMAM-DEN^CYS^ nano-conjugate was determined using transmission electron microscopy (TEM). The TEM images revealed that the PAMAM-DEN / PAMAM-DEN^CYS^ nanoparticles exist as mono-suspension with no aggregation, and the average size of nanoparticles was ~4nm (Fig 1D and 1E).

### Cysteamine-analogue formulation induces autophagy in CF cells

**Fig 2:**
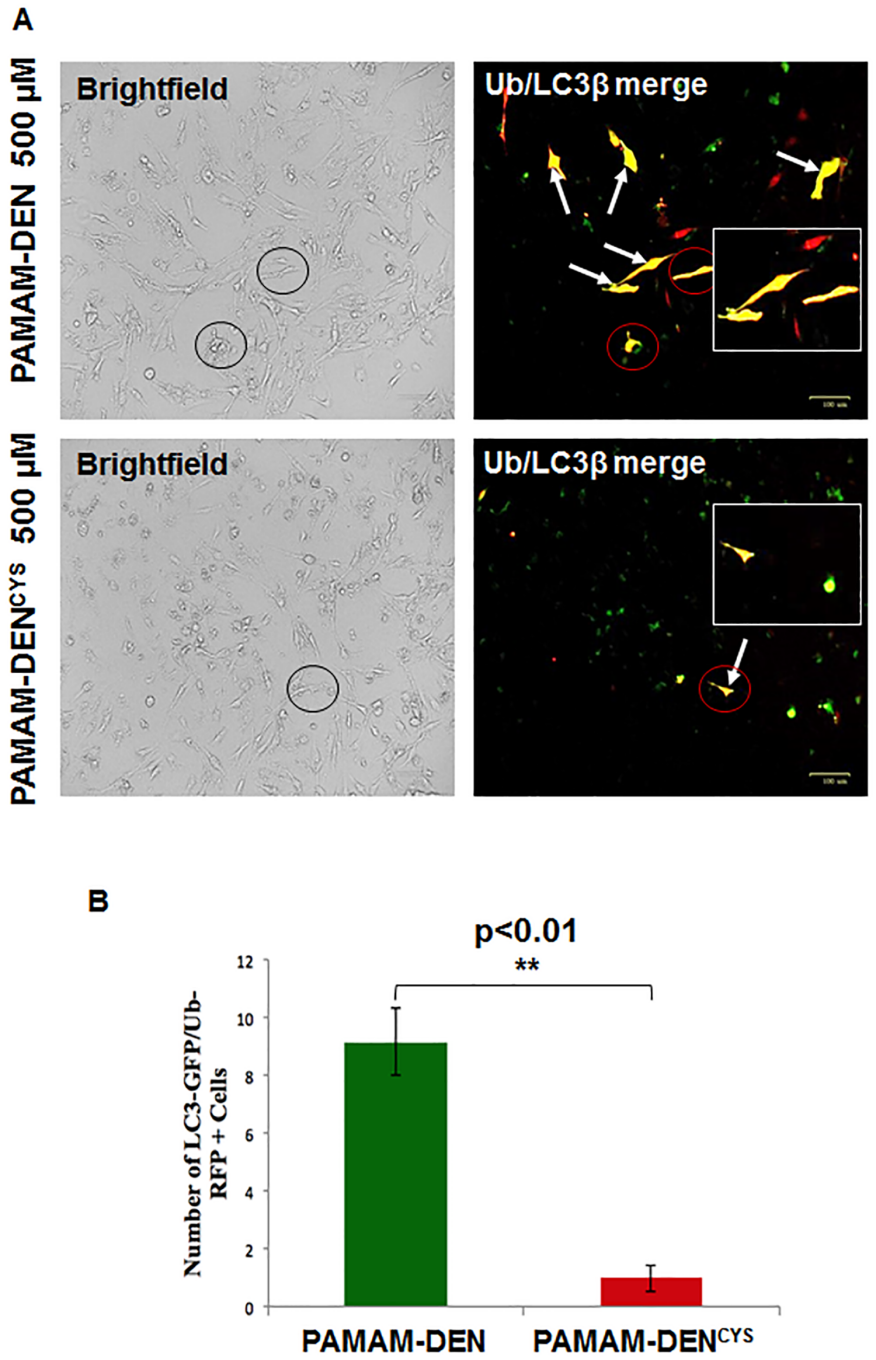
PAMAM-DEN^CYS^ restores ΔF508-CFTR induced autophagy-impairment in CF cells. (A) The IB3-1 cells were co-transfected with RFP-(Ub) Ubiquitin and GFP-(LC3), the autophagy-protein light chain-3 plasmid constructs, and after 24 hrs of transfection, cells were treated with PAMAM-DEN^CYS^ (500μM) and control (PAMAM-DEN). The fluorescence microscopy images were used to evaluate the efficacy of the nano-formulation to restore the intrinsic impaired-autophagy in CF cells. Administration of PAMAM-DEN^CYS^ significantly diminishes the LC3-GFP and Ub-RFP co-localization as compared to control untreated cells. The circles are shown to indicate the location and morphology of the fluorescent cells (red circles) in the respective brightfield image (black circles) to clarify that they are from the same field of view. (B) The data (n = 3, **p<0.01), suggests that PAMAM-DEN^CYS^ could restore autophagy-impairment in CF cells. Scale bar: 100 μm.

In order to develop a drug delivery system capable of allowing sustained and targeted delivery of cysteamine to CF lung epithelial cells, we ‘surface decorated’ G4 PAMAM dendrimers with cysteamine-analogue. We first sought to verify if PAMAM-DEN^CYS^ could rescue the autophagy-impairment in IB3-1 cells [38]. The autophagy reporter assay [18] was used to demonstrate that untreated IB3-1 cells had marked co-localization of autophagosome (LC3-GFP) and proteasome dysfunction (Ub-RFP) markers indicating impairment of autophagy/proteostasis. This co-localization was significantly decreased (p<0.05) in PAMAM-DEN^CYS^ treated cells implicating restoration of autophagy (Fig 2A and 2B). The data suggests that similar to cysteamine, our novel nano-formulation has the potential to rescue ΔF508-CFTR from aggresome-bodies to the PM *via* autophagy-induction, thus providing a strategy for increasing the levels of functional CFTR in CF lungs.

### PAMAM-DEN^CYS^ rescues ΔF508-CFTR from aggresome-bodies and induce plasma membrane trafficking

**Fig 3:**
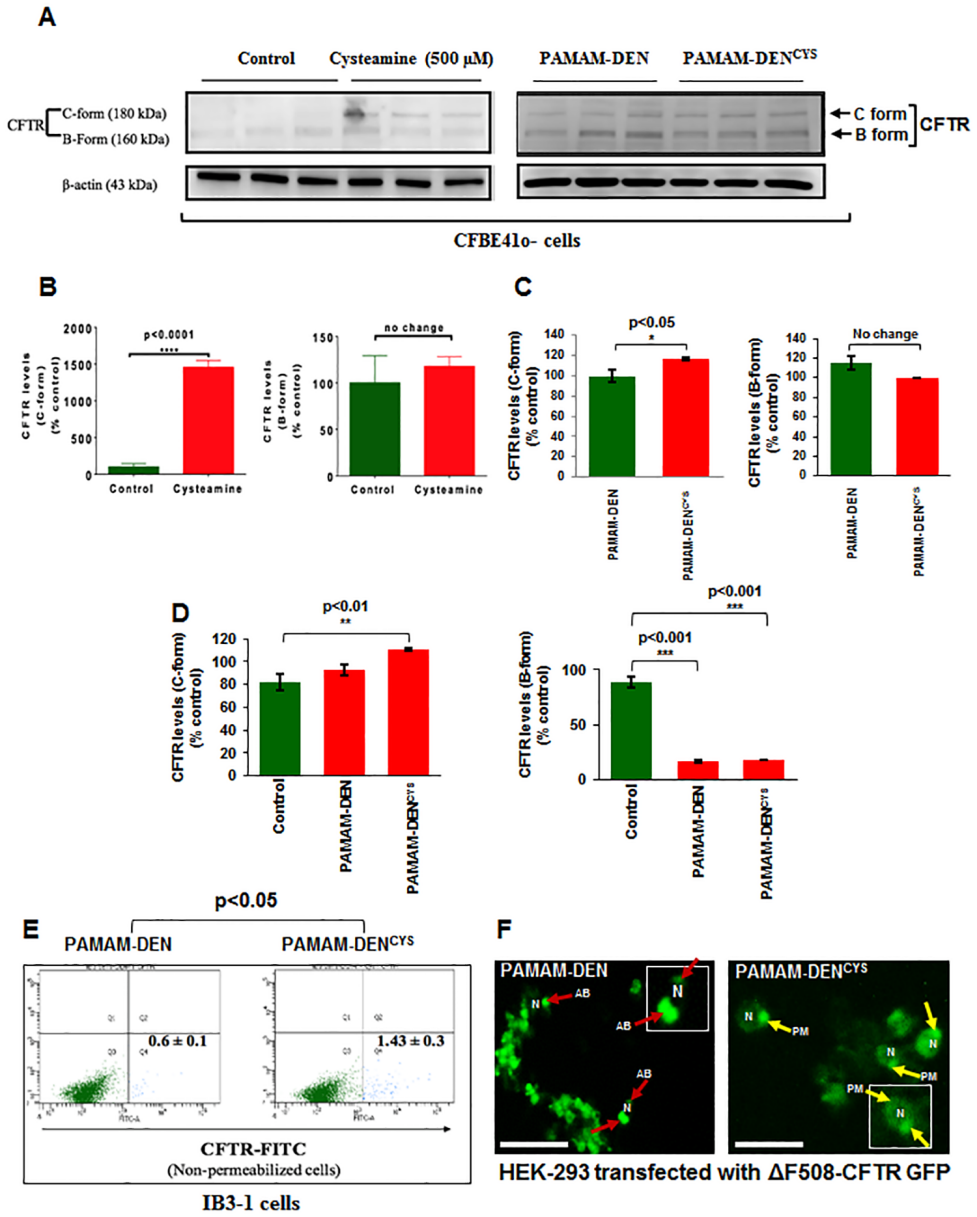
PAMAM-DEN^CYS^ induces trafficking of ΔF508-CFTR to plasma membrane in CF cells. (A, left panel; B) Immunoblotting of total protein extracts from CFBE41o- cells treated with cysteamine (500μM) show significantly (p<0.05) higher protein levels of membrane CFTR (C-form) as compared with untreated control group. (A, right panel; C) Immunoblotting of total protein extracts from CFBE41o- cells treated with PAMAM-DEN or PAMAM-DEN^CYS^ shows a significant increase in ‘C form’ (p<0.05) in PAMAM-DEN^CYS^ treated cells. (D) A comparative densitometry analysis of untreated control, PAMAM-DEN and PAMAM-DEN^CYS^ is shown, although it is more appropriate to compare control vs. cysteamine (B) and PAMAM-DEN vs. PAMAM-DEN^CYS^ (C). Data represent mean ± SEM of triplicate samples. (E) Flow cytometry of non-permeabilized IB3-1 cells treated with PAMAM-DEN^CYS^ show significant (p<0.05) increase in membrane CFTR protein levels as compared to PAMAM-DEN controls. Data represent mean ± SEM of triplicate samples. (F) Immunofluorescence microscopy of HEK-293 cells transfected with ΔF508-CFTR and treated with PAMAM-DEN^CYS^ (500μM, 12 hrs) show increased plasma membrane (PM, yellow arrows) trafficking of CFTR with decreased peri-nuclear aggresome-bodies (AB, red arrows) as compared to controls. To clarify the localization of CFTR, ‘N’ is shown as the nucleus and red arrows show the peri-nuclear aggresome-accumulation of ΔF508-CFTR, while yellow arrows show the membrane-localization of ΔF508-CFTR in PAMAM-DEN^CYS^ treated cells. High-magnification single cell images are shown as insets. Representative image of triplicate samples is shown. Scale bar: 50 μm.

We hypothesized that cysteamine’s efficacy in decreasing CF lung disease will mainly depend on its ability to breach the thick sticky mucus layers in the CF airway, along with maintaining sustained bioavailability of the drug. Therefore, PAMAM-DEN^CYS^ could potentially lead to sustained and targeted therapeutic effect by increasing the access of the drug to CF airway epithelia. We first performed *in vitro* studies to verify that PAMAM-DEN^CYS^ rescues ΔF508-CFTR from aggresome-bodies and induces its membrane trafficking in CFBE41o- cells, thus suggesting its potential for *in vivo* therapeutic application in CF lung disease. We found a significant increase in the ‘C-form’ of CFTR in PAMAM-DEN^CYS^ treated CFBE41o- cells (Fig 3A, right panel; C; p<0.05; PAMAM-DEN vs. PAMAM-DEN^CYS^) as compared to PAMAM-DEN treated control. Intriguingly, we also observed a modest increase in the ΔF508-CFTR ‘C-form’ in PAMAM-DEN-control treated cells, as compared to untreated control cells (Fig 3A, left panel). This could be attributed to the known anti-oxidant properties of these dendrimers [25, 26]. A comparative densitometry analysis of untreated control, PAMAM-DEN and PAMAM-DEN^CYS^ is shown in Fig 3D, although it is more appropriate to compare control vs. cysteamine and PAMAM-DEN vs. PAMAM-DEN^CYS^ (Fig 3B and 3C). We further verified these results by flow cytometry and observed a significant rescue of membrane-ΔF508-CFTR with PAMAM-DEN^CYS^ treatment using non-permeabilized IB3-1 cells (Fig 3E; p<0.05; PAMAM-DEN vs. PAMAM-DEN^CYS^). Moreover, the immunofluorescence microscopy of HEK-293 cells transfected with ΔF508-CFTR-GFP (Fig 3F) showed that PAMAM-DEN^CYS^ is able to rescue the misfolded-ΔF508-CFTR from aggresome-bodies (AB, red arrows, left panel) by inducing its trafficking to the plasma membrane (PM, yellow arrows, right panel). These results provide further evidence that PAMAM-DEN^CYS^ mediated rescue of ΔF508-CFTR to the PM could provide a therapeutic benefit in CF lungs.

### PAMAM-DEN^CYS^ induces bacterial clearance and decreases *Pa* infection in CF cells

**Fig 4:**
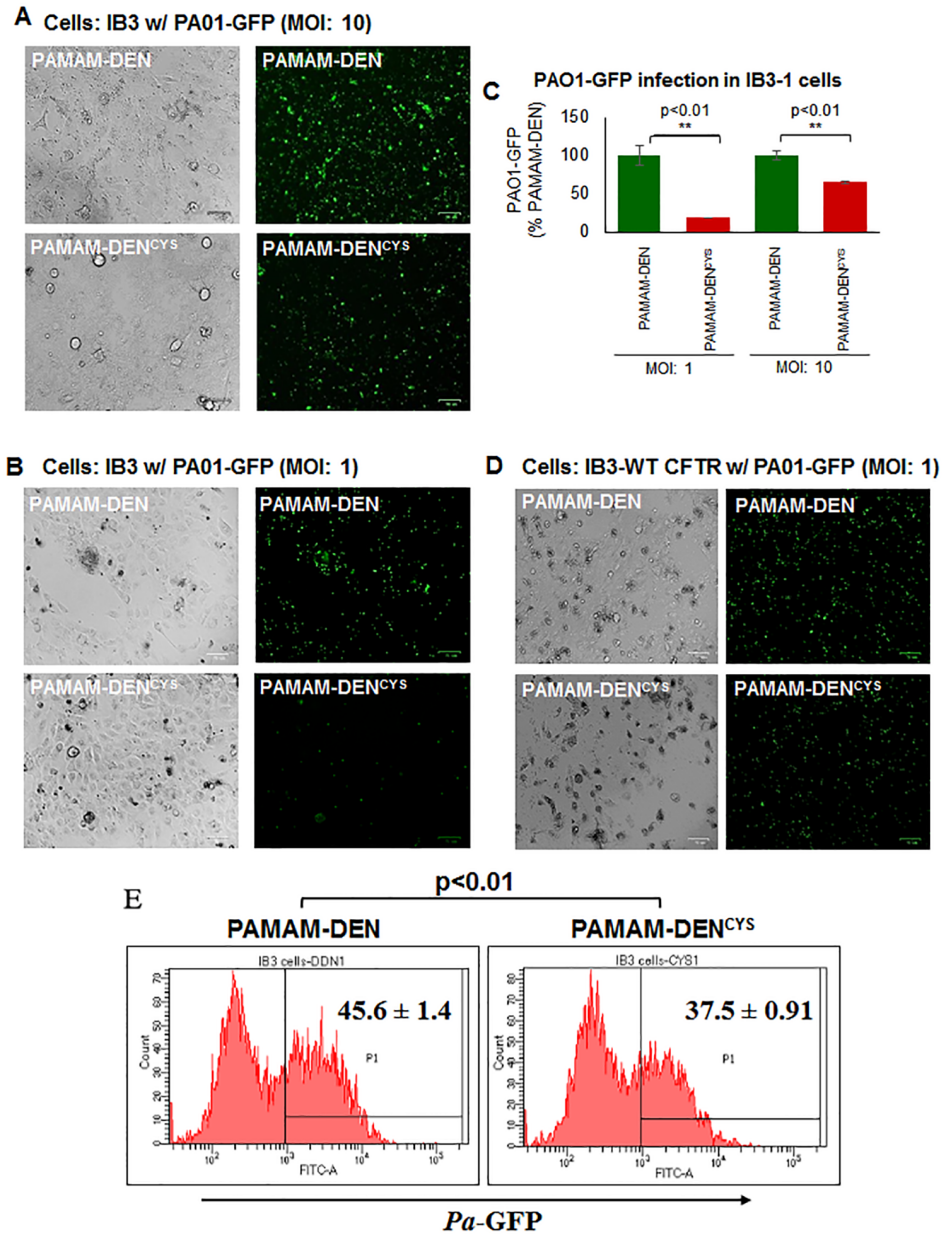
PAMAM-DEN^CYS^ decreases *P*. *aeruginosa* infection in CF cells. (A, B) IB3-1 cells were seeded on a 6-well plate and treated with PAMAM-DEN^CYS^ for 12 hours followed by *Pseudomonas aeruginosa*-GFP (PA01-GFP) infection at MOI of 10 (A) and 1 (B) for 180 minutes. Cells were washed thoroughly with PBS (1x) to remove all extracellular bacteria. Bio-Rad ZOE^™^ Fluorescent Cell Imager was used to capture images. Representative bright field (left) and fluorescent images (right) show cell number and the number of intracellular bacteria respectively. (C) We quantified the number of fluorescent bacteria and found that PAMAM-DEN^CYS^ significantly decreased the *PaO1* counts at both MOI of 1 and 10 (**p<0.01), verifying the efficacy of PAMAM-DEN^CYS^ as an autophagy-inducing antibacterial nano-formulation. (D) IB3-1 cells were transfected with WT-CFTR and infected with PA01 at MOI of 1 as a positive control. (E) In a parallel experiment, IB3-1 cells were infected with PA01-GFP (MOI of 1) for 180 minutes. Post infection, the media was removed and the cells were washed with PBS (1x) and used to quantify the number of intracellular bacteria using flow cytometry based analysis. Analysis of the flow cytometry data shows a significant (*p<0.05) decrease in the number of intracellular bacteria, and provide further evidence substantiating autophagy-mediated bacterial clearance by PAMAM-DEN^CYS^ formulation.

CF patients with ΔF508-CFTR mutation experience chronic *Pa* infections, an important cause of morbidity/mortality in these patients [13]. Based on the known autophagy inducing properties of cysteamine, we postulated that PAMAM-DEN^CYS^ would not only function to restore ΔF508-CFTR to the cell membrane, but also decrease *Pa* infection and thus diminish bacterial virulence in CF airway epithelial cells by autophagy mediated bacterial clearance, in addition to its other anti-microbial effects. To verify this, we assessed the bacterial clearance potential of PAMAM-DEN^CYS^ by treating IB3-1 cells infected with PA01-GFP with PAMAM-DEN and PAMAM-DEN^CYS^, and after thorough washing with PBS (1x) to remove all extracellular bacteria, observed a significant (p<0.01; PAMAM-DEN vs. PAMAM-DEN^CYS^) decrease in intracellular bacterial counts by immunofluorescence microscopy as anticipated (Fig 4A, 4B and 4C). As a control, we transfected IB3-1 cells with WT-CFTR and found that *PaO1* infection did not show a substantial increase in GFP fluorescence, indicating that WT-CFTR supersedes autophagy impairment, which controls *Pa* infection (Fig 4D). The efficacy of PAMAM-DEN^CYS^ was also verified using flow cytometry to quantify the number of intracellular bacteria (Fig 4E). Overall, our data demonstrates autophagy-mediated bacterial clearance by PAMAM-DEN^CYS^, suggesting its potential efficacy in treating chronic CF lung disease, which warrants further investigation using pre-clinical models of CF.

### PAMAM-DEN^CYS^ demonstrates bactericidal and mucolytic properties

**Fig 5:**
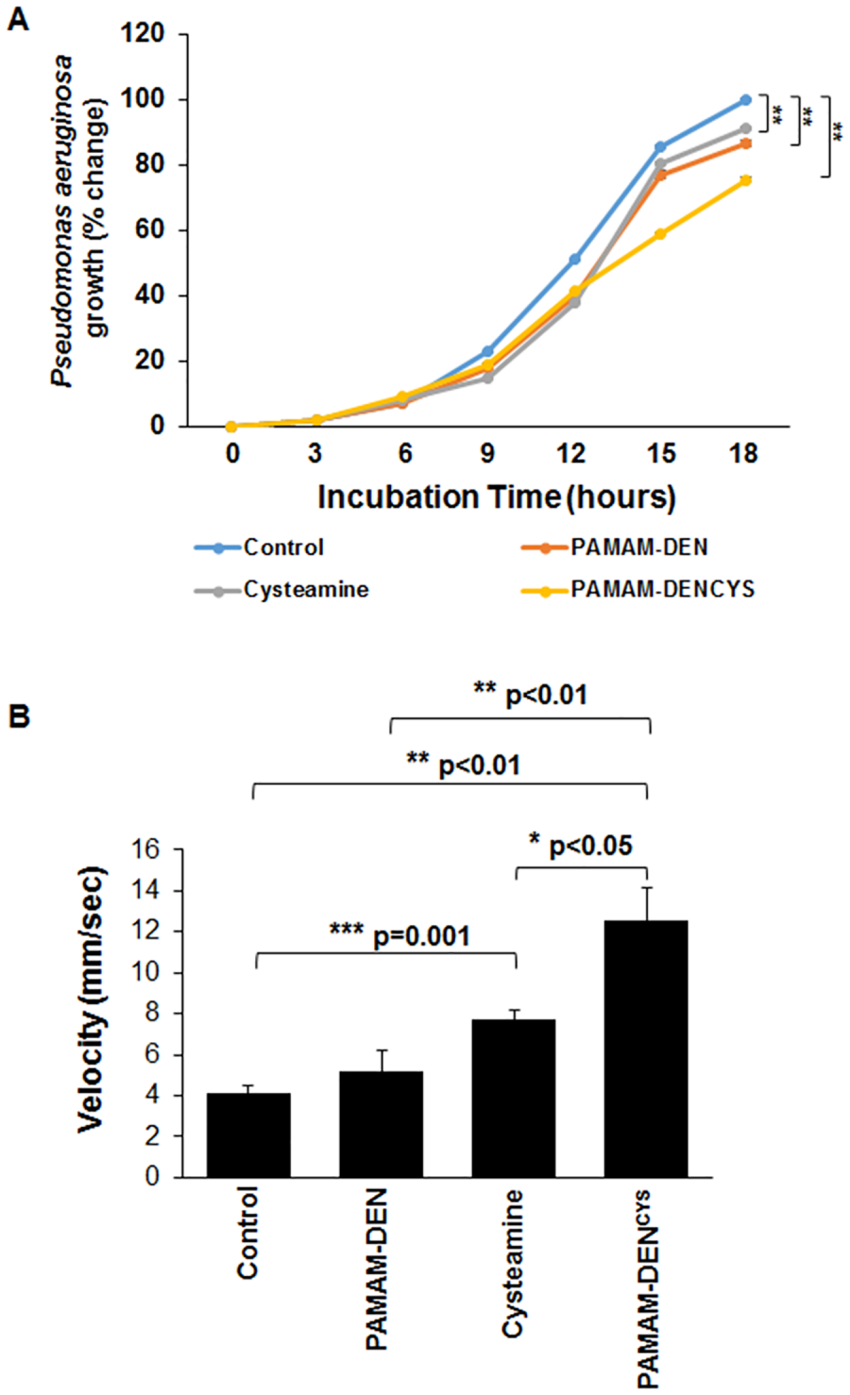
PAMAM-DEN^CYS^ possesses direct bactericidal and mucolytic properties. (A) *Pseudomonas aeruginosa PaO*1 bacteria were incubated in LB broth with either control (PBS), PAMAM-DEN, cysteamine (500 μM) and PAMAM-DEN^CYS^ (500 μM) and the standard growth of bacteria was monitored from 0 to 18 hours by measuring the OD at 625 nm, to assess bacterial growth. We found that PAMAM-DEN^CYS^ significantly (**p<0.01) restricts *Pa* growth as compared to control, PAMAM-DEN or cysteamine treatment groups, with significant differences at 15 and 18 hour time points (**p<0.01). The data suggest that PAMAM-DEN^CYS^ possess direct anti-bacterial activity, which may be one of the several potential benefits of this nano-formulation. The graph represents mean ± SEM, n = 3. (B) The mucolytic activity of PAMAM-DEN^CYS^ was quantified by incubating mucin (5% w/vol) solution with control (PBS), PAMAM-DEN, cysteamine (500 μM) and PAMAM-DEN^CYS^ (500 μM) and measuring their flow rate (velocity, mm/sec) through a 1 ml serological pipette. The data indicates that both cysteamine and PAMAM-DEN^CYS^ have direct mucolytic activity although PAMAM-DEN^CYS^ shows significantly (*p<0.5) better efficacy than cysteamine. Data represents mean ± SEM, n = 3.

Although our data clearly shows that PAMAM-DEN^CYS^ is capable of reducing *Pa* infection in CF cells by autophagy induction, we also wanted to evaluate the direct anti-microbial and mucolytic potential of this nano-formulation. We monitored bacterial survival (growth curve) after incubating the *Pa* culture with control (PBS), PAMAM-DEN, cysteamine (500 μM) and PAMAM-DEN^CYS^ (500 μM). The data indicate that PAMAM-DEN^CYS^ showed significantly (p<0.01) better anti-bacterial efficacy as compared to control, PAMAM-DEN and cysteamine (Fig 5A). We also observed significant (p<0.01) anti-bacterial effect of PAMAM-DEN and cysteamine as compared to control, but this was evident only at 15 and 18 hour time points (exponential phase of growth curve). It is plausible that PAMAM-DEN^CYS^ has improved efficacy as compared to cysteamine (positive control), although further *in vivo* studies are warranted to verify our preliminary findings. We further verified that PAMAM-DEN^CYS^ shows significant (p<0.05) mucolytic activity against porcine mucin, a component of mucus, as compared to cysteamine (Fig 5B). Our data suggests that PAMAM-DEN^CYS^ is a promising nano-formulation for CF treatment, as it can control bacterial infection/growth by inducing autophagy and diminishing components (mucus) essential for their survival *via* its mucolytic effects. The mucolytic affect retained by PAMAM-DEN^CYS^ design (Fig 1A) engineered in this study, is also anticipated to improve drug-delivery and targeting to CF epithelia.

## Discussion

Autophagy-impairment is recently shown to be a critical mechanism involved in pathogenesis of chronic lung diseases, such as cystic fibrosis (CF) and chronic obstructive pulmonary disease (COPD) [2, 7, 8, 18–20, 38]. Moreover, autophagy-inducing drugs have shown potential in diminishing disease pathogenesis in both murine and human studies [2, 18, 20, 21] although challenge is to effectively deliver the drug to CF epithelia through obstructive airway. Hence, in the present study, we developed a dendrimer (PAMAM-DEN)-based cysteamine (PAMAM-DEN^CYS^) analogue that possesses autophagy-inducing and mucolytic properties, and has the potential to rescue ΔF508-CFTR in CF, while also decreasing *Pa* infection that exacerbates CF lung disease (28). Thus, our nano-formulation (PAMAM-DEN^CYS^) has the potential for clinical translation as a novel nano-based therapeutic for ameliorating CF lung disease [28] as discussed below.

Briefly, ΔF508-CFTR mutation and resulting protein misfolding results in a complex dysregulation of multiple cellular processes including autophagy, proteasomal protein processing, and inflammation [8, 39]. This results in premature degradation of ΔF508-CFTR, with a parallel decrease in beclin 1 (BECN1) expression, a protein necessary for autophagy. The cross-linking with constitutively activated transglutaminase 2 (TGM2), [7, 8, 38] decreases beclin 1 expression that results in the formation of perinuclear, ΔF508-CFTR+ aggresome-bodies [8]. Additionally, significant CFTR misfolding and accumulation results in activation of reactive oxygen species (ROS) that induces oxidative stress and inflammation. This ROS activation leads to chronic autophagy-impairment that can promote inflammatory responses and further worsen CF pathogenesis by aggregation of ΔF508-CFTR and other critical ubiquitinated proteins [7, 8, 20, 40]. Therefore, restoration of autophagy is an appealing mechanism to target multiple cellular processes dysregulated in CF.

We and others have tested several autophagy inducers to restore tobacco smoke-induced autophagy-impairment [18, 41, 42]. In this study, we selected cysteamine, a reduced form of cystamine, which is an FDA approved drug for the treatment of cystinosis (22), based on its known anti-oxidant and autophagy inducing activity along with bactericidal, anti-biofilm and mucolytic properties [7], to design a novel nano-formulation for effective delivery to CF lung epithelial cells. While cysteamine has been shown in previous studies to increase membrane-ΔF508-CFTR protein expression, it also has the potential to decrease mucus buildup and control bacterial infection in CF lungs [12]. Although challenge remains to effectively deliver this drug through obstructive airway [40, 43]. Recently, as a proof of concept, an oral form of cysteamine, Lynovex^®^, was used in phase II clinical trials that has shown promise in decreasing acute CF exacerbations in human subjects [44]. Although oral administration may have systemic advantages, we believe that targeted delivery of cysteamine to the CF-epithelia *via* inhalation would have increased therapeutic efficacy by decreasing chronic lung inflammation (anti-oxidant), restoring impaired autophagy (autophagy-inducer) and controlling *Pa* growth/exacerbation (anti-bacterial), the major causes of morbidity and mortality in CF [14, 20, 40, 45]. Indeed, our data shows that PAMAM-DEN^CYS^ is capable of restoring autophagy-impairment in CF cells (Fig 2), while rescuing aggresome-trapped ΔF508-CFTR to the PM (Fig 3). Additionally, we also demonstrate the efficacy of PAMAM-DEN^CYS^ to reduce *Pa* infection in CF cells that act by autophagy-mediated bacterial clearance (Fig 4) and direct bactericidal (Fig 5) mechanisms as discussed. Cysteamine has been shown to possess direct anti-bacterial activity against *Pa* [12, 20]. Intriguingly, our results demonstrate that PAMAM-DEN^CYS^ nano-formulation possess significantly better anti-bacterial activity against *Pa* as compared to cysteamine or PAMAM-DEN/control. Briefly, we observed significantly less bacterial growth in the PAMAM-DEN^CYS^ treatment group compared to control, cysteamine and PAMAM-DEN at 18 hours (Fig 5A). It is conceivable that PAMAM-DEN^CYS^ improves the efficacy of cysteamine by improving bioavailability, thus showing better anti-bacterial effects.

In spite of these benefits, the thick mucus buildup that results from impaired ion transport in the CF lung provides an obstructive physical barrier that diminishes the actual amount of drug delivered to the CF-epithelia. Lately, nano-based drug-delivery strategies have been increasingly utilized in obstructive lung pathologies (such as CF, COPD and asthma) due to its ability to circumvent physical barriers and selectively target disease specific tissues/cells [14, 26, 28, 40, 45]. Some of these studies have used poly-lactide-co-glycolide (PLGA)-based nanosystem that is coated with polyethylene glycol (PEG) to prevent opsonization by the host immune defense and facilitate drug delivery to airway cells [14, 40]. Although PLGA-PEG systems provide effective *in vivo* delivery of encapsulated drug through obstructive mucus [43, 45], we needed the drug to be exposed on the surface in order to retain bactericidal and mucolytic properties of cysteamine. Thus, we utilized PAMAM-DEN^CYS^ which is potentially capable of dissolving the mucus layers thereby facilitating sustained (*via* increased bioavailability) and targeted drug delivery to CF epithelial cells. As a proof of concept, in our preliminary experiments, the PAMAM-DEN^CYS^ nano-formulation demonstrates potent mucolytic property as compared to control, PAMAM-DEN and cysteamine (Fig 5B). Thus, our PAMAM generation 4 (G4) dendrimers with cysteamine bound to terminal ends provides a formulation with mucolytic ability that dissolves the mucus and allows direct absorption of cysteamine by the CF-epithelia. Disruption of airway mucus has multiple positive effects including decreasing mucus-induced inflammation, as well as diminishing a potential colonization site for *Pa*. It remains to be evaluated whether PAMAM-DEN^CYS^ demonstrates a direct anti-biofilm activity against *Pa*, similar to cysteamine [12] which could possibly interrupt the vicious infection cycle that perpetuates CF pathogenesis.

Thus, using a dendrimer-based approach allows noninvasive, targeted administration of a drug (cysteamine) through obstructive and inflammatory barriers and has the potential to reduce CF pathogenesis. Although, many drugs including small molecule correctors (increase CFTR-expression) and potentiators (increase CFTR-function), have shown some potential in rescuing ΔF508-CFTR and decreasing CF pathogenesis, chronic inflammation and hypersecretion of mucus in CF airway remains a challenge by preventing the effective drug delivery to CF epithelia. Our data suggest that dendrimer-based cysteamine formulation would increase cysteamine’s therapeutic potential in CF treatment due to its ability to penetrate through the thick mucus and inflammatory barrier while clearing *Pa*-infection in the lumen of the CF airways.

## Conclusions

Overall, our data provides a preliminary proof of concept that warrants pre-clinical studies to further develop this dendrimer-based nano-formulation (PAMAM-DEN^CYS^) for effectively breaching the difficult to penetrate mucus inflammatory barrier in CF, thereby providing increased bioavailability and targeted airway delivery of a promising CF therapeutic, cysteamine.
